# Can AMH levels predict the need to step up FSH dose for controlled ovarian stimulation following a long GnRH agonist protocol in PCOS women?

**DOI:** 10.1186/s12958-023-01173-8

**Published:** 2023-12-18

**Authors:** Hui Huang, Haijie Gao, Yingying Shi, Bingbing Deng, Xuemei He, Jin Lin, Ping Li

**Affiliations:** 1 Department of Reproductive medicine, Women and Children’s Hospital, School of Medicine, Xiamen University, Zhenhai Road 10, Xiamen, 361000 Fujian China; 2 Xiamen Key Laboratory of Reproduction and Genetics, Xiamen, Fujian China

**Keywords:** Anti-mullerian hormone, PCOS, Ovarian response, rFSH dose, Controlled ovarian stimulation

## Abstract

**Background:**

To explore the role of anti-Mullerian hormone (AMH) in predicting the need to step up recombinant FSH (rFSH) dose following long GnRH agonist protocol in IVF/ICSI cycles of polycystic ovarian syndrome (PCOS) women.

**Methods:**

This is a retrospective cohort study of 825 PCOS women undergoing long GnRH agonist protocol enrolled from Jan 2019 to Dec 2021. The daily rFSH dose at which the first response to rFSH were recorded. The dose at which the first response to rFSH was based on folliculometry during follow up in which two or more follicles reached ≥ 11 mm. A receiver operating characteristic (ROC) curve analysis was done to investigate the ability of AMH to predict the need to step up initial rFSH dose.

**Results:**

PCOS women who needed to step up initial rFSH dose had a significantly higher AMH compared with those didn’t step up initial rFSH dose (11.37 ± 3.25ng/ml vs. 8.69 ± 3.16ng/ml, p < 0.001). In multivariate logistic regression analysis, increased AMH level was an independent factor for the need to step up initial rFSH dose in PCOS patients after adjusted for confounding factors. ROC curve analysis showed AMH could predict the need to step up initial rFSH dose (AUC = 0.738, 95%CI: 0.704–0.773), having 75.4% specificity and 63% sensitivity when the threshold AMH concentration was 9.30ng/ml. 58.8% PCOS women with AMH > 9.30 ng/ml required increased rFSH dose compared to 18.8% of women with AMH ≤ 9.30ng/ml (p < 0.001). Although the clinical pregnancy rate and live birth rate were not significantly different, there was a higher incidence of OHSS among women with AMH > 9.30 ng/ml vs. AMH ≤ 9.30ng/ml (20.8% vs. 15.3%, p = 0.043).

**Conclusion:**

PCOS women with AMH > 9.30 ng/ml were resistant to rFSH stimulation and require increased dose for the cycle recruitment of ovarian follicles.

**Supplementary Information:**

The online version contains supplementary material available at 10.1186/s12958-023-01173-8.

## Background

Polycystic ovarian syndrome (PCOS) is an ovulatory and endocrine disorder affecting 5–20% of women of reproductive age [1]. Ovulation induction or stimulation is the principal infertility treatment for women with PCOS. Usually with a high antral follicle count (AFC), PCOS women are at high risk of developing ovarian hyperstimulation syndrome (OHSS) in in vitro fertilization (IVF) or intra-cytoplasmic sperm injection (ICSI) cycles [2]. A systematic review found an odds ratio of 6.8 for the development of OHSS when polycystic ovarian morphology (PCO) were present [3].

Ovarian stimulation in women with PCOS is challenging as the ovary may produce an uncontrolled response to gonadotropins. A regular starting dose for patients with normal ovarian response may lead to over recruitment and a high risk of OHSS in PCOS patients. However, a low starting dose for less chance of OHSS may lead to unexpected poor response or delayed response [4]. Identifying clinical predictors of a suitable starting dose for individual PCOS patient is necessary.

It is widely accepted that the serum concentration of AMH can be used as a predictor of ovarian response to gonadotrophins during ovarian stimulation. It has been positively associated with the diagnosis of PCOS and a higher risk for the development of OHSS [5]. In women without PCOS, serum AMH has been found to correlate positively with ovarian responsiveness to gonadotrophin stimulation [6, 7]. In PCOS women, associated with a 2- to 3-fold increase in AMH levels [8], the results were the other way around. There were several studies found that PCOS women with markedly raised circulating AMH seem to be resistant to clomiphene citrate or human menopausal gonadotrophin (HMG) ovulation induction and may require a higher starting dose [9–13]. A further study by Ahmed Kamel et al. evaluated predictive factors of the ovarian HMG-responsive dose, defined as the daily dose of HMG to reach follicle development of 11 mm diameter. The result showed that PCOS with AMH > 4.6 ng/ml were resistant to HMG stimulation, requiring dose step up during ART cycles [14]. However, the impact of excess circulating AMH levels on controlled ovarian stimulation by rFSH in women with PCOS has rarely been investigated.

We hypothesized that PCOS women with high AMH levels were resistant to FSH stimulation and could have a delayed response to what had been supposed to be sufficient FSH initial dose. The purpose of our study was to examine whether AMH could predict the need to step up rFSH starting dosage to achieve follicular response for IVF/ICSI in women with PCOS.

## Methods

### Study design and population

This was a retrospective cohort study that used the ART databases from the Department of Reproductive Medicine at Women and Children’s Hospital, School of Medicine, Xiamen University. The study included 825 PCOS who underwent first IVF/ICSI cycle from Jan 2019 to Dec 2021. The inclusion criteria were as follows: (1) age 20–39 years; (2) BMI<30 kg/m^2^; (3) received first IVF/ICSI treatment with the long GnRH agonist protocol; (4) controlled ovarian stimulation with recombinant FSH (rFSH); (5) diagnosed as PCOS according to Rotterdam criteria [15]. All subjects included had two or more criteria which included polycystic ovarian morphology (PCOM), oligo/anovulation, and hyperandrogenism. PCOM was diagnosed by transvaginal ultrasound (≥ 12 follicles in at least one ovary measuring 2–9 mm in diameter and/or an increased ovarian volume of > 10 mL). Oligo/anovulation was defined as cycle interval>6 weeks. Hyperandrogenism was diagnosed either clinically (acne/hirsutism,) and/or biochemically (total testosterone > 0.75 ng/ml). Hirsutism confirmed when modified Ferriman-Gallwey (mFG) score ≥ 5 in Chinese women [16]. The exclusion criteria were diagnosed with any type of endometriosis, or with a history of ovary surgery, any untreated endocrine dysfunction e.g., hypothyroidism/hyperthyroidism, or hyperprolactinemia, and marked biochemical hyperandrogenemia (either Cushing’s syndrome or congenital adrenal hyperplasia). This study was approved by the ethics committee of the Women and Children’s Hospital, School of Medicine, Xiamen University (KY-2022-056).

### Clinical examination and AMH

Clinical examination was done where weight and height were recorded. Basal gonadotropins were drawn on second or third day of menses in regularly menstruating women, or after withdrawal bleed in women with irregular cycles. Serum AMH was drawn from the participants before starting IVF/ICSI cycle, and analyzed using an CLIA kit (Kangrun Biotech, China). The intra- and interassay coefficient of variation of the kit was less than 10% and less than 12%, respectively. The detection range was 0.06-18 ng/mL. All hormone samples drawn in this study were analyzed through immunoassay by Kangrun Kaeser 1000 Immunoassay System (Kangrun Biotech, China).

### Controlled ovarian stimulation and IVF/ICSI

Patients received 3.75 mg long-acting GnRH agonist triptorelin (IPSEN pharma biotech, France) on the day 2–3 of period or 1.25 mg triptorelin on day 20–22 of the cycle with Diane-35. After pituitary desensitization was confirmed (when the E2 level was <25pg/ml, the FSH level was <5 IU/L, the endometrial thickness was ≤ 5 mm and the diameter of part of the antral follicles reached 5-6 mm), controlled ovarian stimulation was commenced. Two different consultants with the same level of training and experience set the starting dose of the rFSH (Gonal-F, Serono, Switzerland). Generally, the starting rFSH dose was based on the algorithm: 100–125 IU/day (BMI<20 kg/m2); >125–175 IU/day (20 ≤ BMI<24 kg/m2); >175–225 IU/day (24 ≤ BMI <30 kg/m2). The individual starting dose could be adjusted 25-50IU according to the age, AMH level and antral follicle count (AFC).

Folliculometry was done by transvaginal ultrasound (TVUS) on the sixth day of rFSH stimulation. Step up of the rFSH was done if the Estradiol (E2) drawn on the sixth day of controlled ovarian stimulation was less than 100 pg/ml, or if the leading follicle was less than 11 mm. The increment of dose was 25-50IU for patients and follow up was repeated 24-48 h later. Continued step up of rFSH was done on sequential follow ups till response was documented.

When at least three leading follicles reached 18 mm, recombinant human chorionic gonadotropin (rhCG) 200-250ug (Ovidrel, Merck Serono, Germany) was used to trigger ovulation. Ovum pick up procedure was done 36-37 h after trigger, and either IVF or ICSI was performed as indicated for all participants. Vaginal administration of progesterone daily (Crione 90 mg, Merck Serono, Germany) was used for luteal support, and serum beta chorionic gonadotropin (β-HCG) was done 2 weeks after embryo transfer to document pregnancy. TVUS was done 2 weeks later to document the presence of intrauterine sac and confirm fetal cardiac pulsations to record clinical pregnancies. Live birth was defined as the delivery of one or more live infants.

### Study variables and outcomes measures

Data concerning age, body mass index (BMI), basal serum follicle-stimulating hormone (FSH), luteinizing hormone (LH), initial starting rFSH dose, dose at which the first response occurred, Gn duration, total dose of rFSH, estradiol level on the day of ovulation trigger, number of oocytes retrieved, clinical pregnancy, and OHSS rate were all recorded. OHSS was classified according to Society of Reproductive Medicine Study Groups of the Chinese Medical Association [17], and its overall incidence was recorded. Based on previous studies [14, 18] and our clinical practice, the dose at which the first response to rFSH was recorded based on folliculometry during follow up in which two or more follicles reached ≥ 11 mm.

### Statistical analysis

The SPSS 20.0 (SPSS Inc, Chicago, IL) software was used for data analysis. Normally distributed measurement data are expressed as the mean ± standard deviation (SD). Two-group comparisons of normally distributed data were performed using the t-test. Measurement data that were not normally distributed were represented by the median (25-75%), and the rank-sum test was used for two-group comparisons. Chi-square test was used to compare categorical data between groups. Multivariate logistic regression analyze was done to estimate the odd ratio (OR) and 95% confidence interval (CI) for the need for increased FSH dose and AMH and other possible confounders. Receiver operating characteristic (ROC) curve was used to determine a cutoff value for serum AMH to accurately predict rFSH dose step up.

## Results

### The baseline characteristics of PCOS women

A total of 825 PCOS women undergoing first IVF/ICSI cycles were collected. Participants were divided into two group according to whether the initial rFSH dose was stepped up to reach the first follicle response. Group A included 508 PCOS patients who did not step up initial rFSH dose, and group B with 317 women who needed to step up initial rFSH dose. Demographic characteristics of participants overall are shown in Table 1. PCOS women who needed to step up initial rFSH dose have higher AFC, BMI as well as serum T concentration. AMH levels were significantly higher among women who needed to step up initial rFSH dose than those who didn’t step up initial rFSH dose (11.37 ± 3.25ng/ml vs. 8.69 ± 3.16ng/ml, P < 0.001). No differences in age, infertility duration, infertility type, or initial rFSH treatment dose were observed between two groups.

**Table 1:** The baseline characteristics of PCOS women grouped according to whether or not the intial rFSH dose were stepped up

	ALL (n = 825)	Did not step up rFSH dose (n = 508)	Need to step up rFSH dose (n = 317)	P Value
Age, y	30.2 ± 3.8	29.6 ± 3.5	29.9 ± 3.4	0.193
BMI, kg/m2	21.26 ± 2.98	21.1 ± 2.8	22.6 ± 3.4	**0.002**
Duration of infertility, y	4.0 ± 2.7	3.9 ± 2.3	4.1 ± 2.4	0.089
Infertility type, %				0.654
Primary	57.4% (473)	57.9% (294)	56.6% (179)	
Secondary	42.6% (352)	42.1% (214)	43.4% (138)	
Serum LH, IU/L	8.18 ± 5.40	8.00 ± 5.44	8.46 ± 5.32	0.237
Serum FSH, IU/L	6.90 ± 1.61	6.98 ± 1.62	6.78 ± 1.59	0.096
Serum T, ng/ml	0.55 ± 0.20	0.53 ± 0.20	0.58 ± 0.21	**< 0.001**
AFC	31.6 ± 9.9	31.0 ± 9.3	32.0 ± 10.8	**0.03**
Serum AMH, ng/mL	9.72 ± 3.45	8.69 ± 3.16	11.37 ± 3.25	**< 0.001**
Initial rFSH dose	112.5 (100, 150)	112.5 (100, 150)	112.5 (100, 150)	0.195

LH: luteinizing hormone; FSH: follicle stimulation hormone; T: testosterone; BMI: body mass index; AFC: antral follicle count; AMH: anti-Mullerian hormone

Data are presented as mean ± standard deviations, percentages (%) or median (25%, 75%). P value comparing two groups are determined with the t- test, the chi-squared test or the Mann-Whitney U test

### Multiple logistic regression analysis for the need to step up initial rFSH dose

A Multiple logistic regression model was established by the status of the need to step up initial rFSH dose and AMH level as well as the possible confounding factors (Table 2). The result showed that an increased chance of stepping up initial rFSH dose was associated with a higher level of AMH (OR = 1.32, 95%CI: 1.23–1.38) and BMI (OR = 1.13, 95%CI: 1.03–1.19) adjusted for age, AFC, FSH, and T levels. This result suggests that AMH may be a predictor of the need to step up the initial rFSH dose.

**Table 2:** Multiple logistic regression analysis for the need to step up initial rFSH dose

	OR	95% CI	*P*
Age, y	1.04	0.99 − 1.09	0.062
BMI, kg/m2	1.13	1.07 − 1.20	< 0.001
AFC	1.00	0.98 − 1.01	0.693
AMH, ng/ml	1.32	1.25 − 1.39	< 0.001
FSH, IU/L	0.95	0.86–1.04	0.265
T, ng/ml	1.56	0.71–3.42	0.256

Data are shown as OR(odds ratios) with 95% CI(confidence interval)

### ROC curve for AMH to predict the need to step up the initial rFSH dose

**Fig. 1:**
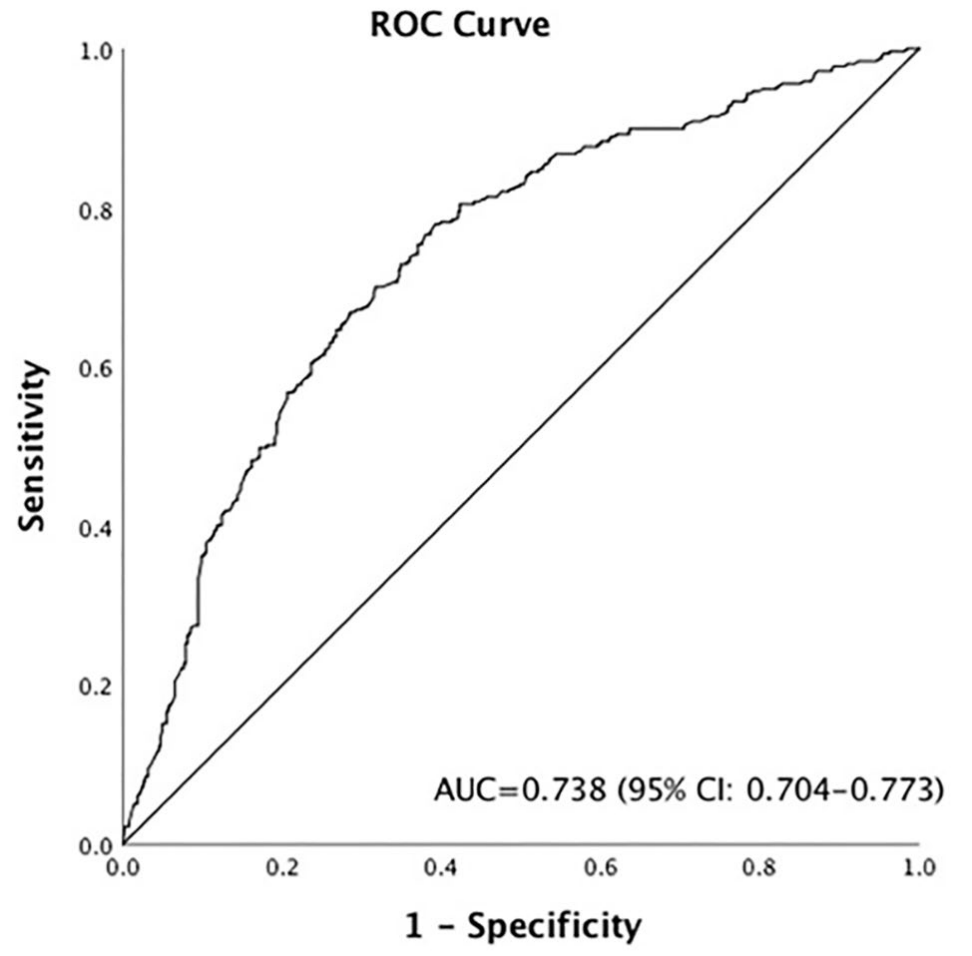
ROC curve showing the accuracy of AMH to predict the need to step up rFSH dose during IVF/ICSI

An ROC curve was drawn to determine a cutoff value for AMH to predict the need for step up of the initial starting rFSH dose. As shown in Fig. 1, the area under the curve (AUC) was 0.738, with 95%CI (0.704–0.773) (p < 0.01), indicating a potential for predicting the need to step up the initial rFSH dose. We took a cutoff value of 9.30ng/ml for AMH at which the sensitivity was 75.4%, and specificity was 63.0%. The chosen cutoff value best represents sensitivity without compromising specificity at the peak of the curve.

### The ovarian stimulation characteristics and pregnancy outcome between AMH ≤ 9.30ng/ml and AMH>9.30ng/ml

With this cut-off (9.30 ng/ml), the outcomes of controlled ovarian stimulation were compared between participants with high AMH vs. low AMH levels. Table 3 highlights that 58.8% PCOS women with AMH > 9.30 ng/ml required step up of the rFSH dose compared to 18.8% of women with AMH ≤ 9.30ng/ml (p < 0.001). Although the clinical pregnancy rate and live birth rate were not significantly different, there was a higher incidence of OHSS among women with AMH > 9.30 ng/ml (20.8% vs. 15.3%, p = 0.043).

**Table 3:** The ovarian stimulation characteristics between AMH≤9.51ng/ml and AMH>9.51ng/ml

	AMH≤9.30ng/ml	AMH>9.3ng/ml	*P*
Cases, n	397	428	
Age, y	29.7± 3.5	29.6± 3.3	0.976
BMI, kg/m2	21.7 ± 3.1	21.7 ± 3.1	0.875
Initial rFSH dose	112.5 (100, 150)	112.5 (100, 150)	0.064
Need to step up rFSH initial dose,%	18.8%(78/415)	58.5%(240/410)	**<0.001**
rFSH dose on first response	137.5(112.5, 150)	150(112.5, 150)	**0.027**
Days of first response	5 (4, 6)	6 (5, 7)	**<0.001**
rFSH duration	9 (8, 11)	11 (9, 12)	**0.026**
Total rFSH dose	1162(1006, 1425)	1393(1171, 1800)	0.064
E2 on day of trigger,pg/ml	3249(2583,4839)	3278(2182,4800)	0.091
Oocytes retrieved	13 (10, 16)	15 (10, 27)	**<0.001**
No. of available embryos	7 (5, 9)	7(5, 9)	0.425
No. of good quality embryos	3 (1,5)	3 (1,5)	0.287
OHSS, %	15.3% (61)	20.8% (89)	**0.043**
Moderate to severe OHSS, %	2.0% (8)	4.2%(18)	0.072
Clinical Pregnancy, %	60.5% (169/279)	56.2% (145/258)	0.304
Miscarriage, %	10.1% (17/169)	11.7% (17/145)	0.445
Live birth, %	53.8% (150/279)	48.8% (126/258)	0.254

OHSS: ovarian hyperstimulation syndrome; E2: estradiol

## Discussion

In this study, we found excessive circulating AMH levels to be ‘negatively’ correlated with ovarian response to rFSH, as PCOS women with serum AMH level above 9.5 ng/ml required step up of initial rFSH dose to reach the follicle recruitment. Furthermore, we demonstrated that PCOS women with higher levels of AMH require higher doses of rFSH and longer duration of stimulation to reach the first ovarian response. In addition, we showed a similar clinical pregnancy rate and live birth rate, but a significantly higher OHSS rates in patients with higher AMH.

The negative effect of high AMH levels on ovarian responsiveness to gonadotrophin may reflect the association between high serum AMH levels and increasing severity of anovulation in PCOS [19]. In vitro studies showed that AMH inhibited the recruitment of follicle growth from the resting primordial follicle pool and attenuated FSH-stimulated preantral follicle growth [20]. Human granulosa cells cultured with high concentration of AMH have shown an inhibition of aromatase activity and reduced follicular response to FSH [21, 22]. It can be conceptualized that high AMH concentrations present in PCOS women is detrimental to the process of folliculogenesis due to its inhibitory influence on the actions of FSH.

These clinical and basic research evidence led us to hypothesis that there is a subgroup of women with PCOS who have higher levels of AMH and who are more resistant to gonadotropin treatment. In this study, we proved that PCOS women with serum AMH level above 9.5ng/ml required step up of rFSH dose during IVF/ICSI cycles. These women were more resistant to ovulation stimulation compared to those with AMH ≤ 9.5ng/ml, requiring more medication and longer duration undergoing stimulation. Although the AUC proved the test to have a certain accuracy in predicting step up of the rFSH, the 75.4% sensitivity for our cutoff value means that 24.6% of those who need a step-up dose would be missed.

Our results are consistent with previous researches done by Amer et al. in 2013 [23] and by Ahmed Kamel et al. in 2017 [14]. Both of them showed that PCOS with markedly raised circulating AMH were resistant to HMG stimulation and might require a higher starting dose. However, the cut off values for serum AMH of 4.7 ng/ml and 4.6ng/ml, respectively, were much lower than that identified by us. Here are several possible explanations. Firstly, we included PCOS women with ovarian suppression with long GnRH agonist rather than GnRH antagonist. Secondly, rFSH was used for ovarian stimulation instead of HMG. Thirdly, different AMH detection kits could result in variation of serum AMH levels. Finally, variable AMH levels across different ethnicities and race may be ascribed to these differences [24]. Therefore, it is important to note that our cutoff AMH level applies only to the AMH kit used in our study and to Asian races. More studies are needed to confirm which value would be more practical for clinical application.

It is of note that factors of AMH, FSH, AFC, age and BMI are combined to predict individualized FSH dosing in controlled ovarian stimulation. These may therefore be confounding factors that could have an effect on responsiveness to rFSH. Multiple logistic regression analysis showed that AMH level and BMI were independent predictors of ovarian delayed response to rFSH treatment adjusted for confounding factors. A recent FSH pharmacokinetic study showed that the FSH and estradiol response in overweight/obese PCOS subjects is lesser than that in lean PCOS following rFSH injection [25]. These results are consistent with our findings that PCOS of higher BMI are resistant to rFSH stimulation and require a higher dose of rFSH. ROC curve analysis showed that the predictive value of BMI was lower than that of AMH for the need to step up rFSH dose (AUC = 0.593, 95%CI: 0.553–0.632, Supplementary Fig. 1). The reason could be that the higher starting doses of rFSH were prescribed for ovary stimulation in women with an increased body mass index.

Although T levels were higher in PCOS patients who needed to step up initial rFSH dose, multivariate regression study showed that T levels were not independently associated with the increasing chance of stepping up initial rFSH dose. Our results are in line with two other studies exploring the association between hyperandrogenism and ovarian response. Kamel et al. suggested that the HMG responsive dose wasn’t associated with androgen levels [14]. A prospective study from Xi et al. showed that the T levels were not different between clomiphene citrate-responsive and clomiphene citrate-resistant PCOS patients [10]. However, another study revealed that PCOS with hyperandrogenism, especially for patients classified as phenotype A (manifested as hyperandrogenism, ovulatory dysfunction, and polycystic ovaries), were more resistant with clomiphene [26]. The role of hyperandrogenism in ovarian response should be further explored by well-designed prospective study and mechanism research.

Women with excessive serum AMH levels are at increased risk of OHSS and a higher risk of cycle cancellation. It was demonstrated that the high E2 level associated with ovarian hyperstimulation had adverse effect on endometrial receptivity, which therefore could compromise pregnancy outcomes in the fresh embryo transfer cycles [27]. Two recent studies found high serum AMH levels were associated with lower live birth rates in women with PCOS [28, 29]. However, our study showed that the clinical pregnancy and live birth rates were comparable between low AMH group(AMH ≤ 9.30ng/ml) and high AMH group (AMH>9.3ng/ml). Our results corroborate the findings of previous meta-analysis of 525 observational studies, reporting poor accuracy for AMH in predicting implantation and clinical pregnancy, even for those with PCOS [30]. Studies mentioned above were all retrospective designed. Further prospective randomized studies and molecular studies are required to clarify the relationship.

The main limitation of this study is its retrospective design. However, the quite large sample size, as 825 consecutive patients fulfilling the study inclusion were included, represent a strength of the study. Furthermore, the initial rFSH dose set was based on two clinicians’ experience. However, the two consultants are of the same level of training and similar clinical experience. The starting dose set by the two consultants are not significantly different, which could reduce bias and enhance the validity of our data.

In conclusion, PCOS with AMH levels above 9.5ng/ml are resistant to rFSH stimulation and may require step up of the rFSH dose during IVF/ICSI cycles. These patients need more medication for follicle recruitment, but at the same time they face a higher risk for OHSS. Our results suggest that PCOS patients with excessive AMH levels may require a relatively high rFSH starting dose, although further prospective randomized studies are needed to identify tailored starting dose without placing them at unnecessary risk of OHSS.

### Electronic supplementary material

Below is the link to the electronic supplementary material.

Supplementary Material 1

Supplementary Material 2

Supplementary Material 3

## Data Availability

All data generated or analysed during this study are included in this published article.

## Abbreviations

rFSH: recombinant follicle-stimulating hormone
PCOS: polycystic ovarian syndrome
AMH: anti-Mullerian hormone
ROC: receiver operating characteristic
CI: confidence intervals
OR: odd ratio
AUC: area under the curve
AFC: antral follicle count
OHSS: ovarian hyperstimulation syndrome
IVF: in vitro fertilization
ICSI: intra-cytoplasmic sperm injection
ART: assisted reproductive technology
HMG: human menopausal gonadotrophin
GnRH: Gonadotrophin-Releasing Hormone
LH: luteinizing hormone
BMI: body mass index
E2: Estradiol
Gn: Gonadotropin
