# Supplementary figures and images for: Can AMH levels predict the need to step up FSH dose for controlled ovarian stimulation following a long GnRH agonist protocol in PCOS women?
Source: Reprod Biol Endocrinol. 2023 Dec 18;21:121. doi: 10.1186/s12958-023-01173-8 (PMC10726541; doi:10.1186/s12958-023-01173-8)

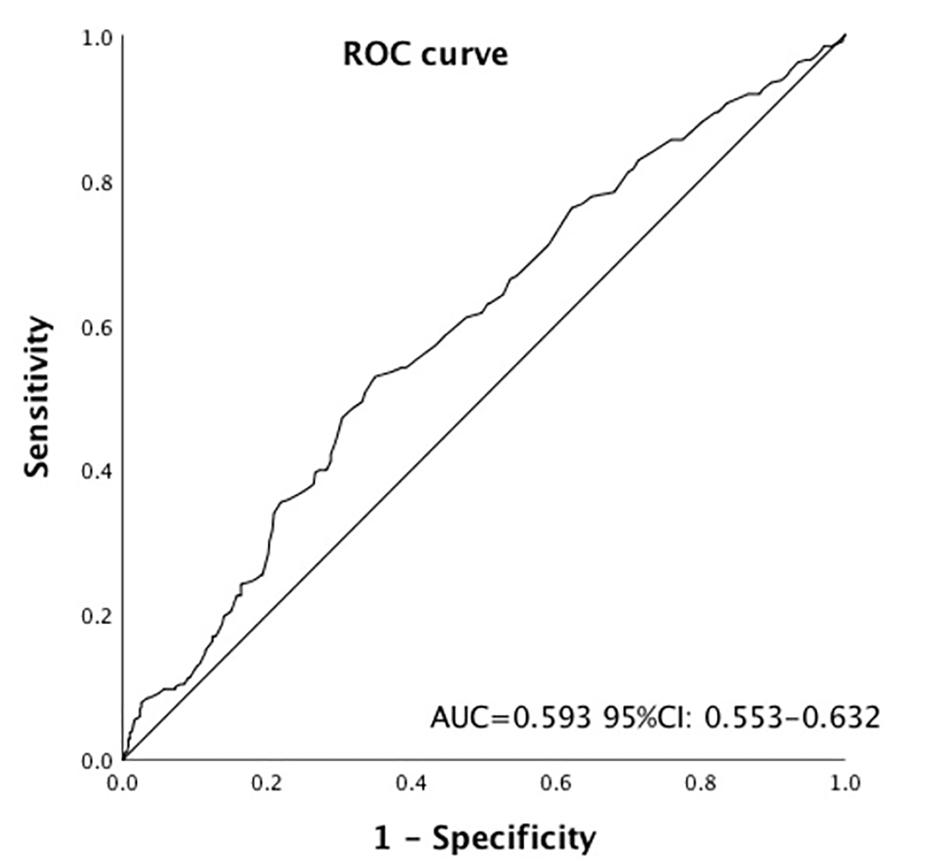

Supplement: Supplementary file 2 — Supplementary Material 2 [file 12958_2023_1173_MOESM2_ESM.png]
